# Academic dermatology chair and chief characteristics

**DOI:** 10.1016/j.ijwd.2021.08.003

**Published:** 2021-08-08

**Authors:** Alyssa M. Thompson, Swetha Atluri, Danielle Yee, Kyla N. Price, Jennifer L. Hsiao, Vivian Y. Shi

**Affiliations:** a University of Arizona, College of Medicine, Tucson, Arizona; b University of California Los Angeles–Olive View, Department of Medicine, Sylmar, California; c University of Illinois, College of Medicine, Chicago, Illinois; d University of California Los Angeles, Department of Medicine, Division of Dermatology, Los Angeles, California; e University of Arkansas for Medical Sciences, Department of Dermatology, Little Rock, Arkansas

**Keywords:** Department chair, division chief, academic medicine, academia, gender, dermatology

**What is known about this subject in regard to women and their families?**

• Gender gaps in academic dermatology exist.
• Very few studies have explored characteristics and academic achievement among academic dermatology leaders.

**What is new from this article as messages for women and their families?**

• On average, female leaders in dermatology have lower publication counts and h-indexes and receive less funding from the National Institutes of Health than their male counterparts.
• Achievements and gender disparities in research metrics increase with academic rank promotion.
• Gender gaps in leadership positions in academic dermatology appear to be narrowing.

Alt-text: Unlabelled box

Dear Editors,

Department chairs and division chiefs (DCs) are respected leaders in academic medicine. Data on characteristics and academic achievement among dermatology DCs are limited. Herein, we examine the demographics of U.S. dermatology DCs and identify scholarly merit benchmarks.

In April 2020, all dermatology residency programs were identified using the Accreditation Council for Graduate Medical Education Public Advanced Program Search. Departmental/division websites and the worldwide web were used to record DC demographics. The National Institutes of Health (NIH) Research Portfolio Online Reporting Tool, Scopus database, and h-index determined NIH funding, publication records, and citation impact, respectively. The 2019 Blue Ridge Institute for Medical Research determined the top 20 NIH-funded (20-NIH) dermatology departments/divisions.

## Publications

**Fig. 1:**
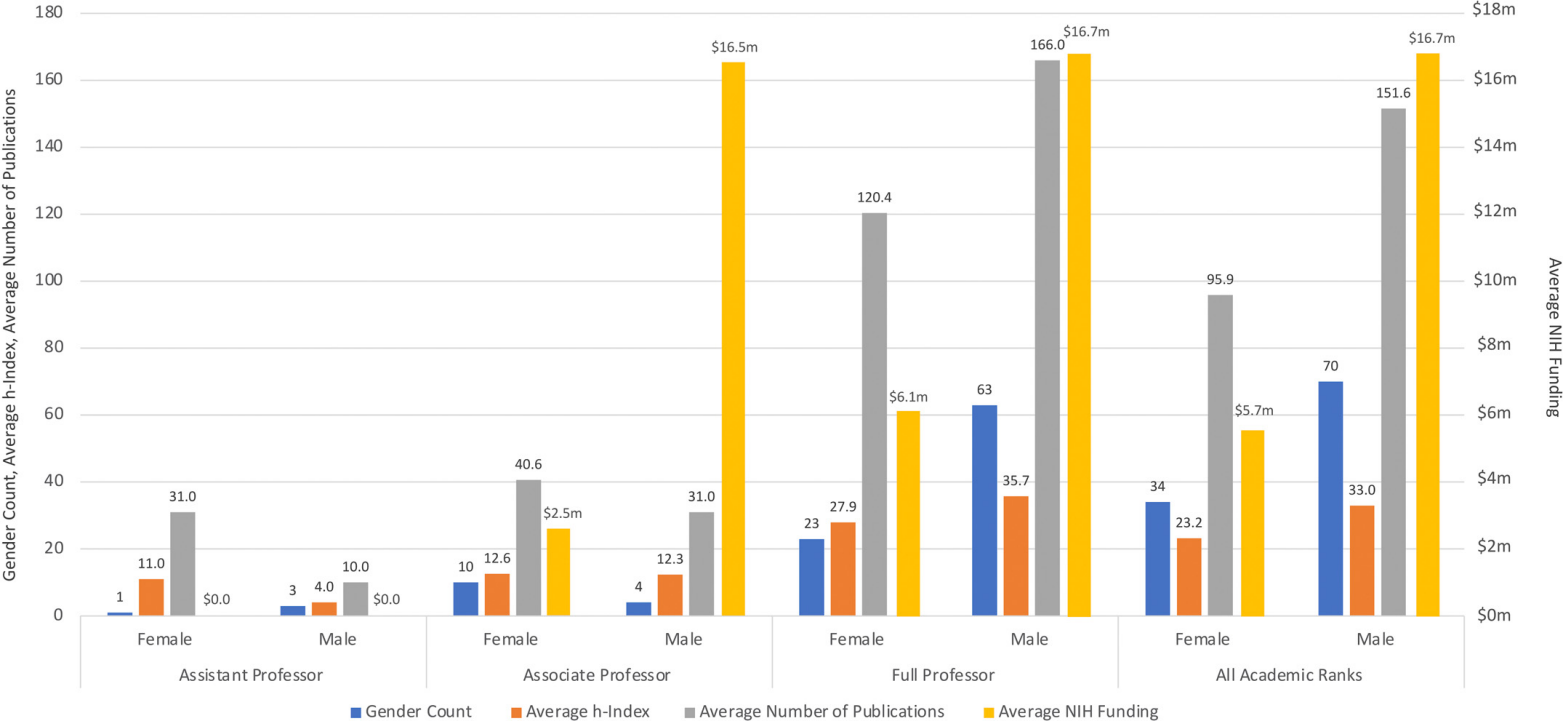
Scholarly merits of dermatology chairs and chiefs by academic rank.

The characteristics of the residency programs and DCs are shown in Table 1. DCs have an average of 122 publications (137 men, 89 women), MD/PhDs have 155 (197 men, 71 women), MDs have 121 (134 men, 95 women), and DOs have 17 (22 men, 1 women). The mean publication count is 134 (152 men, 96 women), and is the highest for full professors (n = 154; 166 men, 120 women; Fig. 1). DCs of the 20-NIH had 69% more publications than non-20-NIH (n = 184; 177 men, 207 women vs. n = 109; 128 men, 72 women).

**Table 1:** Dermatology chairs and chief demographics

	Men	Women	Both
**Total chairs and chiefs, n (%)**	82 (67.2)	40 (32.8)	122 (100)
**Total filled resident positions, mean (range; standard deviation)**			
Male DC	―	―	12.0 (3–29; 5.3)
Female DC	―	―	13.4 (3–29; 6.1)
**Leadership title (n = 122), n (%)**			
Department chair	63 (67.0)	31 (33.0)	94 (77.1)
DC	19 (67.9)	9 (32.1)	28 (23.0)
**Academic title (n = 122), n (%)**			
Full Professor	63 (73.3)	23 (26.7)	86 (70.5)
Associate professor	4 (28.6)	10 (71.4)	14 (11.5)
Assistant professor	3 (75.0)	1 (25.0)	4 (3.3)
Unknown	12 (66.7)	6 (33.3)	18 (14.8)
**Interim (n = 122), n (%)**			
Yes	4 (50.0)	4 (50.0)	8 (6.6)
No	78 (68.4)	36 (31.6)	114 (93.4)
**Concurrently residency program director (n = 122), n (%)**			
Yes	14 (58.3)	10 (41.7)	24 (19.7)
No	68 (69.4)	30 (30.6)	98 (80.3)
**Degree (n = 122), n (%)**			
MD	65 (65.7)	33 (33.3)	99 (81.1)
MD, PhD	12 (70.6)	6 (35.3)	17 (13.9)
DO	5 (83.3)	1 (16.7)	6 (4.9)
**AAD Fellow (n = 122), n (%)**			
Yes	80 (68.4)	37 (31.6)	117 (95.9)
No	2 (40.0)	3 (60.0)	5 (4.1)
**Affiliated with a top-20 NIH-funded institution (n = 122), n (%)**			
Yes	15 (75.0)	5 (25)	20 (16.4)
No	67 (65.7)	35 (34.3)	102 (83.6)
**Merits, mean; median (range; standard deviation)**			
Number of publications (n = 120)	137.4; 86 (1–953; 153.7)	89.2; 69 (1–481; 90.5)	121.7; 75.5 (1–953; 137.9)
H-index (n = 120)	30.5; 22 (1–108; 24.8)	21.5; 18 (0–67; 15.5)	27.6; 21 (0–108; 22.6)
First publication year (n = 120)	1990; 1987 (1968–2018; 11.4)	1993; 1992 (1977–2018; 10.8)	1991; 1989 (1968–2018; 11.3)
Total NIH funding 1985–2020 (n = 44)	$15.2m; $4.5m ($722–$76.7m; $19.3m)	$5.3m; $2.5m ($0.2m–$17.9m; $6.3m)	$12.7m; $4.4m ($722–$76.7m; $17.4m)
Number of NIH grants (n = 46)	46.7; 32 (1–159; 48.0)	22.4; 13 (4–62; 18.1)	40.9; 22.5 (1–159; 43.8)

AAD, American Academy of Dermatology; DC, division chief; m, million; NIH, National Institutes of Health

Programs that did not have a DC listed on the department/division website were excluded. AAD fellow status was recorded from the AAD member search. The top-20 NIH-funded dermatology departments ranked from 1 to 20 (Blue Ridge Institute for Medical Research): Yale University, University of Alabama at Birmingham, Stanford University, University of Pennsylvania, Northwestern University at Chicago, New York University School of Medicine, University of Michigan at Ann Arbor, University of California at Davis, University of California San Francisco, University of Pittsburg at Pittsburg, John Hopkins University, Duke University, Case Western Reserve University/Cleveland Clinic Lerner COM, Oregon Health & Science University, University of Colorado Denver, Boston University Medical Campus, University of California San Diego, Columbia University Health Sciences, University of Miami School of Medicine, and University of Southern California.

## H-index

DCs have an average h-index of 28 (31 men, 22 women); MD/PhDs have 37 (45 men, 20 women), MDs have 27 (29 men, 23 women), and DOs have 8 (11 men, 0 women). The mean h-index for DCs with academic titles is 30 (33 men, 23 women) and is highest for full professors (34; 36 men, 28 women). DCs of the 20-NIH have nearly two-fold higher mean h-indices (46; 48 men, 38 women vs. 24; 27 men, 19 women).

## National Institutes of Health funding

Between 1985 and 2020, DCs received an average of $13 million ($15 million for men, $5 million for women) in cumulative NIH funding overall, MD/PhDs received $17 million ($19 million for men, $10 million for women), MDs received $11 million ($14 million for men, $4 million for women), and DOs lacked funding. Among DCs with academic titles, the average NIH funding was $14 million overall ($17 million for men, $6 million for women) and was highest for full professors ($14 million; $17 million for men, $6 million for women). On average, DCs of the 20-NIH received 55% more funding ($17 million; $19 million for men, $8 million for women vs. $11 million; $13 million for men, $4 million for women).

Overall, DCs have strong research merits; however, there are twice as many men. Achievements and gender disparities in DC research metrics increase with rank promotion, which is dependent on duration in academics and affiliation with the 20-NIH. On average, female DCs have lower numbers than their male counterparts in all metrics analyzed. Gender gaps may be narrowing: A study found that 26.1% of North American dermatology chair/director/head positions were filled by women (Shah et al., 2018), versus 32.8% in our study.

Our findings corroborate a prior study on dermatology faculty that found similar demographic and research metric gender discrepancies (Shah et al., 2018). Between 2015 and 2019, 61.0% of all NIH funding for dermatology research was awarded to men, and male dermatology chairs earned a 9.7% higher median salary in 2018 (Price et al., 2021; Sachdeva et al., 2020). Several systematic gender-based disadvantages may be contributing to these gaps (Lewiss et al., 2020).

Study limitations include a confounding variable of time spent in rank and possible reverse causality between obtaining leadership position and research merits. Limitations from the Accreditation Council for Graduate Medical Education and departmental websites include reliance on accuracy. The Blue Ridge ranking report does not show historic data, inhibiting trend analysis. The NIH Research Portfolio Online Reporting Tool only searches for the principal investigator and queries from 1985 onward.

Aside from scholarly merits, successful DC traits also include strong leadership, interpersonal skills, and a vision for their institution. Increased mentorship and holistic career development may narrow gender gaps.

The results demonstrate that, despite receiving significantly less NIH funding, female DCs have achieved h-indices and publication numbers not far behind those of their male counterparts. The receipt of NIH funding is in part subjective. Given the much lower level of funding received by female DCs, their level of productivity is remarkable, because the h-index is the most objective measure of impact of the research output.

## Funding

None.

## Study approval

N/A

## Conflicts of interest

None.
